# Muscle regeneration in dystrophin-deficient *mdx *mice studied by gene expression profiling

**DOI:** 10.1186/1471-2164-6-98

**Published:** 2005-07-13

**Authors:** R Turk, E Sterrenburg, EJ de Meijer, G-JB van Ommen, JT den Dunnen, PAC 't Hoen

**Affiliations:** 1 Center for Human and Clinical Genetics, Leiden University Medical Center, Wassenaarseweg 72, 2333 AL Leiden, Nederland; 2 Leiden Genome Technology Center, Leiden University Medical Center, Wassenaarseweg 72, 2333 AL Leiden, Nederland; 3 Department of Physiology and Biophysics, Howard Hughes Medical Institute, University of Iowa, 400 Eckstein Medical Research Building, Iowa City, IA52240-1101, U.S.A

## Abstract

**Background:**

Duchenne muscular dystrophy (DMD), caused by mutations in the dystrophin gene, is lethal. In contrast, dystrophin-deficient *mdx *mice recover due to effective regeneration of affected muscle tissue. To characterize the molecular processes associated with regeneration, we compared gene expression levels in hindlimb muscle tissue of *mdx *and control mice at 9 timepoints, ranging from 1–20 weeks of age.

**Results:**

Out of 7776 genes, 1735 were differentially expressed between *mdx *and control muscle at at least one timepoint (p < 0.05 after Bonferroni correction). We found that genes coding for components of the dystrophin-associated glycoprotein complex are generally downregulated in the *mdx *mouse. Based on functional characteristics such as membrane localization, signal transduction, and transcriptional activation, 166 differentially expressed genes with possible functions in regeneration were analyzed in more detail. The majority of these genes peak at the age of 8 weeks, where the regeneration activity is maximal. The following pathways are activated, as shown by upregulation of multiple members per signalling pathway: the Notch-Delta pathway that plays a role in the activation of satellite cells, and the Bmp15 and Neuregulin 3 signalling pathways that may regulate proliferation and differentiation of satellite cells. In DMD patients, only few of the identified regeneration-associated genes were found activated, indicating less efficient regeneration processes in humans.

**Conclusion:**

Based on the observed expression profiles, we describe a model for muscle regeneration in *mdx *mice, which may provide new leads for development of DMD therapies based on the improvement of muscle regeneration efficacy.

## Background

Duchenne muscular dystrophy (DMD) is caused by mutations in the gene encoding dystrophin, a subsarcolemmal protein functioning within the dystrophin-associated glycoprotein complex (DGC)[1,2]. This complex connects the intracellular cytoskeleton to the extracellular matrix. The DGC is concentrated at the Z-lines of the sarcomere and confers the transmission of force across the muscle fibre[3]. Disruption of this link results in membrane instability, which eventually leads to sarcolemmal ruptures[4,5]. Influx of extracellular calcium alters molecular processes like muscle contraction and activates proteolytic activity. Affected muscle fibres become necrotic or apoptotic, and release mitogenic chemoattractants, which initiate inflammatory processes [6-8]. Cycles of degeneration and regeneration eventually lead to irreversible muscle wasting and replacement by fibrotic and adipose tissue.

Muscle has the potential to regenerate by activation of undifferentiated myogenic precursor cells (satellite cells), which are normally quiescent and situated between the basal membrane and the myofibers[9,10]. Upon activation, satellite cells proliferate and divide asymmetrically, with the daughter cells having divergent cell fates[11]. Only one of the daughter cells differentiates, progresses towards the myoblast-stadium, and subsequently fuses with other myoblasts or with damaged muscle fibres to induce muscle fibre repair. The other daughter cell remains in a proliferating state or returns to quiescence[12]. Genetic mutations responsible for DMD are also present in satellite cells. Hence, the ability to restore normal muscle function remains obstructed. A small number of muscle fibres are able to produce functional dystrophin, mostly due to secondary mutations in myogenic precursor cells which restore the reading frame[13]. However, these so-called revertant fibres are in a too small minority to alleviate the pathology of the dystrophin-deficiency. Exhaustion of the satellite cell pool due to degeneration and regeneration cycles is thought to critically contribute to the disease[14].

The *mdx *mouse model for DMD has a spontaneous mutation in exon 23 of the Dmd gene, introducing a premature stopcodon[15,16]. The pathology of the *mdx *mouse is characterized by histologically well-defined stages with similarity to the human pathology. Neonatal muscle tissue appears to be unaffected. Necrotic or apoptotic processes in combination with inflammation emerge at approximately 3 weeks of age[15]. Regeneration processes are initiated around the age of 6 weeks and continue while alternating with ongoing degeneration until 12 weeks of age [17-19]. Contrary to the lethal human pathology, the *mdx *mouse somehow recovers from the progressive muscle wasting, and does not show the accumulation of connective and adipose tissue[17,20]. However, *mdx *mice do show a decline in their regeneration capacity at advanced age (>65 weeks), while necrotic processes persist[21]. Since the degeneration processes are similar to those seen in human pathology, the regenerational differences may hold one of the clues of restoration of proper muscle function.

Although previous studies have studied gene expression levels in the *mdx *mouse [22-27], regeneration processes were not studied in full detail. We studied the regeneration process through genome-wide monitoring of gene expression levels[28] in healthy control and *mdx *mice at 9 time points from 1 to 20 weeks of age, while putting emphasis on time points where regenerative activity is maximal (6–12 weeks), a period which was not analysed in detail in a previous time course study[23]. According to the temporal gene expression profiles, we determined which pathways are active during regeneration with respect to normal muscle aging. The majority of identified genes presented in this study have not been described before and provide a substantial addition to the elucidation of the temporal phasing of degeneration and regeneration. By careful annotation based on existing literature, new light is shed on the pathology and subsequent recovery in the *mdx *mouse. Furthermore, we compared gene expression profiles to those of human DMD patients and found only modest overlap in regeneration-associated genes. This confirms that regeneration is no longer an active process at the age at which the patients were profiled (5–12 years old).

## Results and discussion

### Global comparison of *mdx *and control mice

Gene expression levels were determined in hindlimb muscle tissue from *mdx *and control mice at 9 time points, ranging from 1 to 20 weeks. Differential gene expression levels were calculated per time point by subtraction of the average normalized intensities of control samples from those of the *mdx *samples to correct for normal aging processes. The effects of the normal aging processes on gene expression are relatively minor, and are discussed below. Statistical significance was calculated per time point by performing a Student's *t*-test. Differential gene expression was considered significant when p-values were lower than 0.05 after applying a Bonferroni correction for multiple testing (p ≤ 6.43 × 10^-6^). Out of 7,776 temporal gene expression profiles 1,735 were selected, which satisfied the significance criterion at one or more time points [see Additional file 1].

**Figure 1:**
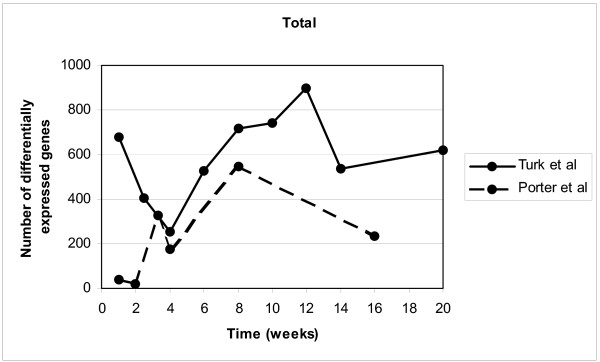
**Amount of differentially expressed genes**. The number of statistically significantly differentially expressed genes between *mdx *and control mice measured across 9 consecutive timepoints from 1 to 20 weeks in this study (continuous line) are compared to the number of differentially expressed genes found in the study of Porter *et al.*[27] (dashed line).

The number of differentially expressed genes per time point changes considerably during the time course, an effect also shown in a previous study by Porter *et al.*[27] (Figure 1). The number of differentially expressed genes peaks at the age of 8–12 weeks, coinciding with the period of maximal muscle regeneration. Interestingly, also at the first two time points (1 and 2.5 weeks of age), where the histology of the *mdx *muscle is not different from that of control mice, a large number of genes was differentially expressed, indicating differences in muscle development in dystrophin-deficient animals. The majority of these genes (553/677 and 355/407, respectively) also show statistically significant differences in expression at later time points. This overlap can be explained by the assumption that the repertoire of gene products used for muscle growth and development also functions in muscle regeneration.

In this report we will describe the expression changes of two main categories in more detail: genes coding for proteins within the costamer and the dystrophin-associated glycoprotein complex (DGC), and genes involved in regeneration.

### The Dystrophin-Glycoprotein Complex

**Figure 2:**
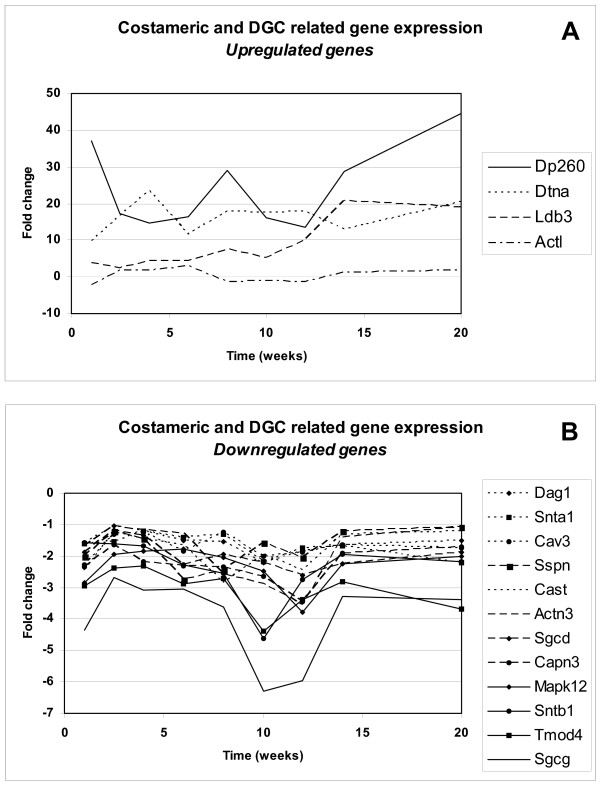
**Costameric and DGC related gene expression**. Fold change in gene expression levels between *mdx *and control muscle tissue measured across 9 consecutive timepoints from 1 to 20 weeks of costameric and DGC related genes. Figure 2A shows the fold changes of the statistically significantly upregulated genes over time; Figure 2B shows the downregulated genes.

The effect of dystrophin-deficiency on expression levels of dystrophin-glycoprotein complex (DGC)-related genes, or genes with associated functional relevance within the costamer has not been reported in previous gene expression profiling studies [22-26], with the exception of the study of Porter and co-workers[27]. In the Porter study, a downregulation of dystrophin was reported, but no changes in gene expression of other components of the DGC. A selection of 52 genes was made based on an overview by Ervasti *et al. *of members of the costameric protein network[29] (Additional file 4). According to the statistical selection criteria, 4 genes were upregulated (Figure 2A) and 12 were downregulated (Figure 2B) in the *mdx *mouse. Although a decrease in dystrophin expression was found in our study, the stringent statistical criteria were not met.

#### Upregulated DGC related genes

We find that the retina-specific isoform of dystrophin (Dp260) is expressed in skeletal muscle of *mdx *mice, whereas Dp260 cannot be detected in hindlimb muscle of control mice. Expression of Dp260 was detected by an oligonucleotide probe within the unique first exon of this transcript. The promoter of the Dp260 isoform resides in intron 29, downstream of the *mdx *mutation (exon23). Transgenic *mdx *mice, which overexpress Dp260 via an alpha-actin promoter, show a restoration of a stable association between costameric actin and the sarcolemma, a re-assembly of the DGC, and an overall alleviation of the pathology[30]. Increased transcription initiation of Dp260 might therefore be a natural adaptation for the lack of the muscle specific isoform of dystrophin. However, in contrast to the artificially raised expression by the alpha-actin promoter, the expression of Dp260 in the *mdx *mouse through the original promoter does not seem to be strong enough to compensate for loss of the full-length muscle specific isoform.

We found a continuous upregulation of alpha-dystrobrevin (Dtna) gene expression with maximum differential expression at 4 weeks in *mdx *mice. Dystrobrevin is a phosphotyrosine-containing protein localized at both the sarcolemma and the postsynaptic side of the neuromuscular junction (NMJ), where it binds to either dystrophin or utrophin [31-34]. Dtna has been described to function as a signalling mediator within the DGC[34]. Transcription of Dtna is activated when myoblasts differentiate into multinucleated myotubes[35]. Newey *et al. *reported that Dtna-protein levels are significantly reduced in the *mdx *mouse at the sarcolemma, whereas the protein level was unchanged at the NMJ. This would be consistent with a stabilizing action of Dtna upon binding to dystrophin or utrophin, since dystrophin is not present at the sarcolemma, whereas utrophin is expressed at the NMJ. They proposed a model, where localized translation of Dtna transcripts contributes to synapse formation[36]. Upregulation of Dtna in the *mdx *mouse might indicate an attempt to compensate for the increased turnover of the protein, in order to stabilize the post-synaptic side of neuromuscular junctions of affected muscle fibres, and retain neuronal connection.

Our results show a continuous upregulation of LIM domain protein 3 (Ldb3, Cypher/ZASP). Studies in Ldb3 knock-out mice demonstrated that ablation of Ldb3 eradicates the structural integrity of the Z-line in contracting striated muscle and causes a severe form of congenital myopathy[37]. Upregulation of Ldb3 indicates the necessity for stabilization of the Z-line in *mdx *mice, compensating the undermining effect of dystrophin-deficiency.

#### Downregulated DGC related genes

It can be seen that gene expression levels of several core-proteins of the DGC, e.g. the transmembrane proteins dystroglycan (Dag1), sarcospan (Sspn), and two members of the sarcoglycan-complex (Sgcd, Sgcg), are lower in *mdx *mice, over the whole time course. Lower expression levels were also detected in other members of the sarcoglycan complex (Sgca, Sgcb, Sgce) and in dystrophin (Dmd, oligonucleotide at the 3' end), but these were not statistically significant. The decrease in expression of DGC-related genes was most prominent during regeneration (8–12 weeks).

Interestingly, DGC related gene expression levels restore to pre-regeneration levels subsequent to the regeneration period, but remain lower than normal (control) level. Similarly, protein levels of core-proteins of the DGC have been shown to be severely reduced in dystrophin-deficient *mdx *mice[38]. It is suggested that the secondary displacement of DGC core-proteins is due to a decrease in protein synthesis and/or assembly, or due to an increase in protein degradation. Similarly, in sarcoglycan-deficiencies the absence of a single subunit causes the loss or strong reduction of the entire sarcoglycan protein complex [39-43]. Since our study reveals a downregulation of mRNA levels of the DGC core-proteins, we conclude that alterations in transcriptional activity also contribute to the decrease in protein levels. As transcription of members of the DGC is likely to be co-ordinately regulated[44], downregulation of these members as seen in *mdx *mice can occur via inhibition or downregulation of shared transcriptional activators.

### Regeneration

In the *mdx *mouse, regeneration of affected muscle tissue is most prominent at the age of 6–12 weeks, after which a stabilized condition is reached. To identify pathways active in regeneration, we studied five categories of differentially expressed genes covering major functional characteristics of regenerative tissues (trophic factors, proteases, membrane associated proteins, signal transduction, and transcription, Additional file 5). This selection of 166 genes was typed for temporal effects during regeneration, and the pathways to which they belong. Since signal transduction pathways are still poorly annotated in current genomic databases, these pathways were constructed from the literature. In our study, a pathway is only considered activated or repressed, when multiple members show differential gene expression.

#### Temporal effects during regeneration

**Figure 3:**
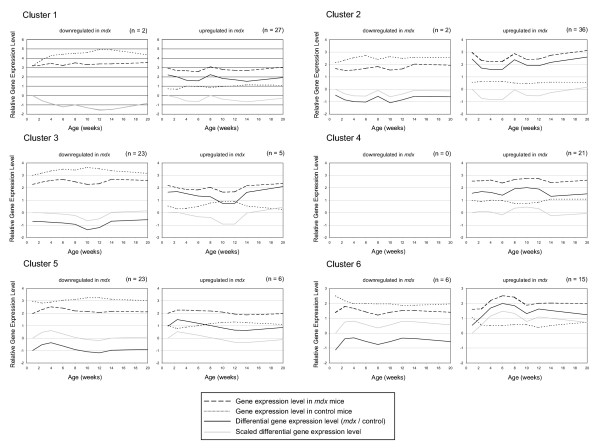
**Temporal effects during regeneration**. K-means clustering (k = 6) classifies gene expression profiles according to similarity in temporal patterns based on the scaled differential gene expression levels (grey line). For each cluster the up- and downregulated genes are shown separately. Unscaled differential gene expression levels are shown (black line), which are representative for the ratio between the *mdx *gene expression levels (dashed line) and control gene expression levels (small dashed line). Relative gene expression levels are obtained after normalization and coincide with the natural logarithm.

Differential gene expression profiles, based on the ratio between *mdx *and control mice, were scaled to the first time point. Differential expression profiles can therefore be compared independent of the ratio level, which enables the detection of temporal effects. Using k-means clustering (k = 6), differential gene expression profiles were classified according to their temporal similarity. The unscaled temporal effects of *mdx *and control gene expression profiles are shown per cluster for the up- and downregulated genes separately (Figure 3). Genes, which show an upregulation in gene expression during the regenerative phase, are present in clusters 1 (n = 27), cluster 2 (n = 36), and cluster 4 (n = 21). The temporal effect is determined by the gene expression profile of the *mdx *mouse, since gene expression is continuously low without temporal changes in the control mouse. Downregulation of gene expression in the *mdx *mouse during regeneration is primarily seen in cluster 3 (n = 23). During normal aging, which can be seen in the control mouse, gene expression increases until the age of 10 weeks, followed by a slow decrease. During the regenerative phase in the *mdx *mouse, however, the expression of these genes is downregulated markedly.

#### Notch-Delta pathway

**Figure 4:**
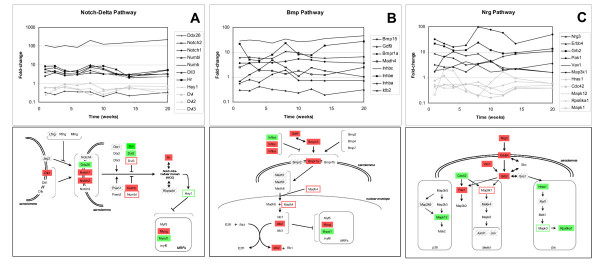
**Reconstruction of active regeneration pathways**. Regeneration-associated pathways were constructed based on differentially expressed genes, literature study and gene ontology. Expression levels of genes in the Notch-Delta pathway (Panel A), the Bmp15 pathway (Panel B), and the Neuregulin3 pathway (Panel C) are plotted as fold-changes between *mdx *and control mice, as a function of age. In the pathway diagrams, filled boxes refer to upregulation (red) and downregulation (green) at 8 weeks. Outlined boxes refer to upregulation (red) and downregulation (green) at other timepoints than 8 weeks. Shaded grey boxes represent genes which are not detected, or are not represented on the microarray. White boxes represent genes that show no differential expression between *mdx *and control mice.

**Figure 5:**
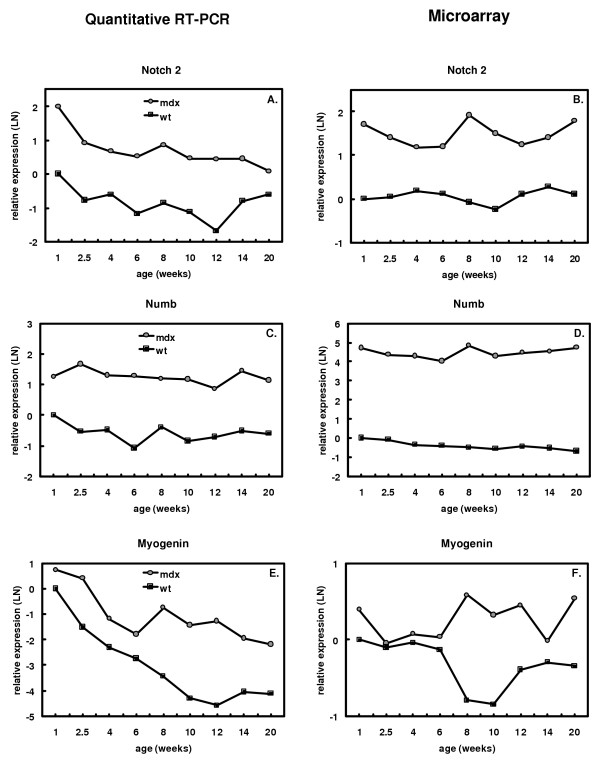
**Confirmation of microarray data for genes in the Notch-Delta pathway by quantitative RT-PCR**. Expression levels of Notch2 (Panel A and B), Numb (Panel C and D), and Myogenin (Panel E and F) in *mdx *(grey circles) and wild-type (black squares) mice at 1 to 20 weeks of age were measured by quantitative RT-PCR (Panel A, C, E) and expression microarrays (Panel B, D, F). Expression levels relative to those in 1 week-old wild-type mice are plotted on a logarithmic scale (natural logarithm).

Gene expression levels of a number of genes functioning in the Notch-Delta pathway are upregulated (Notch1, Notch2, Hr), whereas others (Dxd26, Dvl, Dvl2) are downregulated in the *mdx *mouse at 8 weeks of age (Figure 4A). The gene expression of Dll3 and Numb are switched on in the *mdx *mouse, while no gene expression can be detected in the control mouse (Supplemental Table 1 [see Additional file 4]). The differential expression of the upregulated genes is mostly increased during the regeneration period (6 to 12 weeks) (Figure 4A). For several genes in the Notch-Delta pathway, quantitative RT-PCR experiments were performed to confirm the temporal expression profiles found on the microarray. In accordance with the microarray results, quantitative RT-PCR experiments demonstrated higher expression of Notch2, Numb and myogenin in *mdx *than in control mice at all ages (Figure 5).

Previous work by Conboy *et al.*[11] indicates the role of the Notch-Delta signalling pathway in the regulation of proliferation versus differentiation of asymmetrically dividing satellite cells by Notch or Numb, respectively. According to Delfini *et al.*[45], Notch is expressed in immature myoblasts, while Delta (Dll) expressing cells are more advanced in myogenesis (post-mitotic myoblasts and muscle fibres). Notch activation is thought to inhibit transcription factors containing a basic helix loop helix domain (bHLH) [46-50], via the induction of Hairy and Enhancer of Split 1 (Hes1)[51], thereby inhibiting myogenic differentiation. Numb-expressing cells are able to undergo myogenic differentiation, because the Notch-Delta pathway is inhibited (Figure 4A). Based on our results, the Notch-Delta signalling pathway, notably the expression of Notch or Numb, is responsible for the determination towards proliferation or differentiation of activated satellite cells in the *mdx *mouse. Since gene expression profiling detects proliferation and differentiation processes simultaneously, satellite cell activation and commitment are ongoing, parallel processes.

#### Bmp pathway

Various members of a Bmp-associated pathway (Bmp15, Gdf9, Bmpr1a, Madh4, Inhbc, Inhbe, Inhba, and Idb2) are differentially expressed in the *mdx *mouse (Figure 4B). The gene expression of Bmp15, Bmpr1a, Inhbc, Inhbe, and Idb2 is switched on in the *mdx *mouse, while expression cannot be detected in the control mouse. Bone Morphogenetic Protein 15 (Bmp15) is a member of the transforming growth factor-β (TGF-β) family. Bmp15 induces transcription of Inhibitors of DNA Binding proteins (Idb1-3) via binding to Bone Morphogenetic Protein Receptor type I (Bmpr1a), and the downstream transportation of Smad-complex (Madh8-Madh4) to the nucleus[52,53]. Idb-proteins function as positive regulators of cell growth by binding to Retinoblastoma 1 (Rb1). This leads to the activation of the E2f transcription factor, which plays a role in cell-cycle regulation. Furthermore, Idb-proteins inhibit myogenic differentiation through binding to MRFs[54]. The activation of Idb2 in the pre-regeneration period is indicative of an inhibition of the myogenic differentiation. This inhibition seems to be alleviated during the regeneration period by a decrease in differential expression of Idb2. Although most of the differentially expressed genes in the Bmp pathway are continuously upregulated, the expression of a number of genes peaks during regeneration (Inhbc, Inhbe, Bmp15, and Gdf9)(Figure 4B). The Inhibin proteins (Inhbc/e), likely to be antagonists of Bone Morphogenetic Proteins[55], are also upregulated. Altogether, this points to a positively and negatively controlled regulation of the Bmp15 pathway. Our data suggests that the Bmp15 pathway has an important function in the balancing of proliferation and differentiation of myoblasts, necessary for effective upscaling of muscle-mass.

#### Neuregulin pathway

In our study we found that several members of the Epidermal Growth Factor-like (EGF-like) Neuregulin pathway are differentially expressed (Figure 4C). The signalling cascade is activated by the binding of Neuregulin3 (Nrg3) to the extracellular domain of the upregulated protein tyrosine kinase v-erb-a erythroblastic leukemia viral oncogene homolog 4 (ErbB4)[56]. Both Nrg3 and ErbB4 are expressed in the *mdx *mouse and cannot be detected in the control mouse. The interaction between Nrg3 and ErbB4 activates epidermal growth factor-like signal transduction via binding of the adaptor protein Growth factor receptor bound protein 2 (Grb2), which peaks at the initiation of regeneration (Figure 4C). Grb2 can activate Mitogen activated kinase kinase 1 (Map3k1)[57], whose differential gene expression is increased at 2.5 weeks as well as during regeneration. The activation of the MAP kinase pathways eventually leads to transcriptional induction (reviewed in[58]) through members of Activating protein complex 1 (Ap1), like Jund1 and Jun.

Depending on the protein complexes formed, specific transcription activation will lead to different biological processes ranging from proliferation to differentiation. Furthermore, we find that other Grb-interacting proteins like Vav 1 oncogene (Vav1)[59], and p21-activated kinase 1 (Pak1)[60] are switched on and upregulated, respectively. The differential gene expression of Vav1 increases during the initiation of regeneration, and might play a role in the clustering of integrins for cell adhesion[61]. Pak1 differential gene expression is increased during regeneration. Downstream genes activated by Pak1 regulate cytoskeletal dynamics, proliferation and cell survival signalling[60]. Furthermore, our results show that the Erk pathway is downregulated in the *mdx *mouse as well as the p38 pathway.

### Comparisons with other studies

In contrast to previously published studies of temporal gene expression profiling in the *mdx *mouse [25-27], we primarily focus on regeneration. The majority of differentially expressed genes in regeneration (148 out of 166) in our study have not been reported as differentially expressed in the *mdx *mouse in other studies. Apart from important differences in gene coverage (the Affymetrix U74v2 GeneChips used in the other studies lack probe sets for 22/166 genes), we explain the limited overlap by differences in cut-off levels: as we applied very stringent statistical tests, we could avoid setting a cut-off level for the fold change, thereby picking up genes with small but consistent fold changes, which can be biologically very relevant, especially in the case of transcription factors. This may also explain the large difference between the study of Porter *et al. *and our study in the number of genes found differentially expressed at the early timepoints (Figure 1), where mainly subtle expression changes are expected.

Goetsch *et al. *reported results from a gene expression profiling studies during muscle regeneration induced by cardiotoxin injection in wildtype mice[62]. The authors concluded that muscle regeneration is a complex process that requires the coordinated modulation of the inflammatory response, myogenic precursor cells, growth factors, and the extracellular matrix for complete regeneration of muscle architecture. A similar study of cardiotoxin-induced muscle regeneration, recently published by Zhao and Hoffman, reported that embryonic positional cues (Wnt, Shh, and Bmp) were not induced, whereas expression of factors involved in satellite cell proliferation and differentiation (MRFs, Pax, Notch1, and FGFR4) was recapitulated[63]. Our study, which also asserts satellite cell activation, proliferation, and differentiation, shows differences in muscle regeneration between *mdx *and wildtype mice. Bmp and EGF-like signalling pathways are activated during regeneration in the *mdx *mouse, as well as upregulation of members of the Notch-Delta pathway. In contrast to the upregulation of Pax7 in wildtype mice, we have found upregulation of Pax3.

Altogether, these findings suggest that dystrophin-deficiency might lead to enhanced regeneration processes in hindlimb muscles over and above those found in wildtype mice. It is likely that the regeneration pathways identified in our study are also active in the *mdx *diaphragm, given that the expression of their downstream targets, the muscle regulatory factors myf-5, myoD, and myogenin, are even more elevated in *mdx *diaphragm than in hindlimb[64]. As demonstrated in another recent study[65], the regeneration capacity per se is high and does not explain why the muscle wasting in the *mdx *diaphragm is more severe than in the hindlimb. Other factors such as higher workload and different involvement of the immune system are likely to contribute.

To discern active processes between the lethal human and regenerative murine dystrophin-deficiency, the selected murine gene expression profiles at 8 weeks of age were compared to those of DMD patients[66]. Out of 166 regeneration-associated transcripts, 19 genes could be detected that are differentially expressed in both the human and murine muscular dystrophy (Additional file 5). Seven of these overlapping genes showed an opposite differential expression between human DMD and *mdx*, of which Platelet derived growth factor beta (Pdgfb) and Paired box 3 (Pax3) are discussed below.

Gene expression of Pdgfb is upregulated in the *mdx *mouse (18.6 fold), where it is downregulated (-1.7-fold) in DMD patients. In the *mdx *mouse, Pdgfb shows an increase in gene expression during regeneration (present in cluster 4, Figure 3). Pdgfb was immunolocalized in infiltrating macrophages, regenerating muscle fibres, and myofibre nuclei of affected dystrophic muscle tissue[67]. The mitogen Pdgfb stimulates myoblast proliferation, while inhibiting myoblast differentiation[68]. It seems to have a similar role during regeneration. Paired box 3 (Pax3) gene expression is activated in the *mdx *mouse relative to the control. Its gene expression increases during regeneration, peaking at 12 weeks of age, while hPax3 is downregulated in DMD patients (-1.6-fold). Pax3 is capable of activating the expression of the muscle regulatory factors Myod1, Myf5 or Myogenin, and thereby activating the myogenic program[69].

The limited amount of overlapping genes between *mdx *mice and human DMD patients, as well as a number of genes showing opposite expression (i.e. Pdgfb and Pax3), suggests that processes active in regenerating mouse muscle are not active in human patients at the time gene expression was profiled (Age: 5–12 years old). This corresponds with clinical findings that patients older than 5 years have surpassed active regeneration processes[70]. The discovery of genes showing opposite regulation may partly explain the differences in regeneration efficiency and lethal manifestation of the pathology between dystrophin-deficient human and murine muscles.

## Conclusion

**Figure 6:**
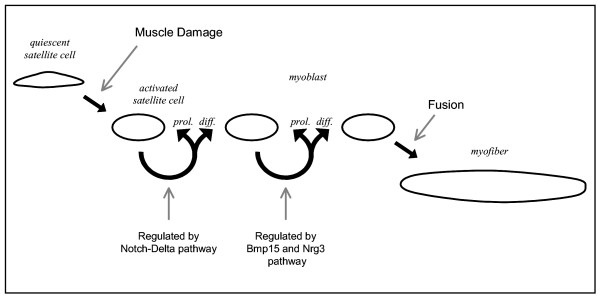
Schematic model of processes in regeneration.

*Mdx *mice lack a functional DGC at the sarcolemma. As a consequence, gene expression of most DGC members is downregulated. *Mdx *mice suffer from massive muscle fibre necrosis starting at the age of 3 weeks. Regenerative processes, starting approximately at the age of 6 weeks, largely restore muscle tissue architecture, although muscle fibres remain centrally nucleated. Recovered muscles of *mdx *mice have slightly diminished strength and higher fatigability. By analysing temporal expression profiles in *mdx *and control mice, we have identified genes and pathways involved in regeneration. The expression of these genes peaks between the ages of 6–12 weeks. Based on the observation that several of these genes are not expressed in control muscle and based on gene ontology classification and further literature annotation, we suggest a role for these genes in activation, proliferation, or differentiation of satellite cells and myoblasts. We propose the following model (Figure 6). Muscular dystrophy leads to muscle fibre necrosis, which attracts inflammatory cells, and release of trophic factors. These factors activate quiescent satellite cells, which as a consequence start to proliferate and differentiate. This divergent cell-fate is controlled by the Notch-Delta pathway. Activated satellite cells differentiate to myoblasts, which proliferate and differentiate as well. The balance between these cell-fates may be regulated by the level of Numb and the activation of Bmp15 and Nrg3 signalling pathways. Differentiation of myoblasts eventually leads to fusion with affected muscle fibres or to the formation of new muscle fibres. The genes and pathways active in regeneration are reminiscent of embryonic myogenesis. We hypothesize that the newly formed or repaired muscle fibres are similar to those in the pre-necrotic phase, but are more able to adapt to dystrophin-deficiency through remodelling of muscle structure and fibre composition. Since many regeneration-related genes remain higher expressed in *mdx *than in control muscle, it seems that regeneration processes are active throughout the life span of the animal. Regenerative processes appear to be most effective when mice reach adulthood, and normal growth processes cease. Regeneration and muscle development are both dependent on the availability of satellite cells, and these processes will therefore compete for the satellite cell availability when activated simultaneously. In the human situation, regeneration processes seem to be exhausted before growth is finished. Together with the accumulation of fibrotic and adipose tissue, exhaustion is thought to be the reason of the lethal manifestation of the disease in human patients. Prolongation of the regenerative capacity by activating the described pathways and/or replenishment with a pool of 'regeneration-primed' cells may therefore provide an attractive strategy in the treatment of muscular dystrophy.

## Methods

### Target preparation and hybridisation

Hindlimb muscle tissue was isolated from *mdx *(C57Bl/10ScSn^mdx/J ^(Jackson) × C57Bl/6NCrl (Charles River) × CBA/JCrl (Charles River)) and control (C57Bl/10ScSnOlaHsd, Harland) at the ages of 1, 2 1/2, 4, 6, 8, 10, 12, 14, and 20 weeks (2 individuals per time point per strain). Total RNA was isolated as described previously (Turk *et al.*, submitted). cRNA was prepared by linear amplification and concurrent incorporation of amino-allyl UTP, followed by chemical coupling to monoreactive Cy3 or Cy5 dyes[71]. Labelled targets (1.5 μg cRNA per target) were hybridised overnight on prehybridised murine oligonucleotide microarrays (65-mer with 5'-hexylaminolinker, Sigma-Genosys mouse 7.5K oligonucleotide library, printed in duplicate) using an automatic hybridisation station (GeneTac, Genomic Solutions). Posthybridisation washes were performed as described previously[71].

### Data analysis

Hybridisations were performed in a dye-swap fashion using temporal loop-designs [72-74], enabling optimal detection of gene expression differences between adjacent time points for both *mdx *and control targets [see Additional file 2] Microarrays were analysed by GenePix Pro 3 feature extraction software (Axon). Local background-corrected, median spot intensities were normalized simultaneously for all microarray experiments using Variance Stabilization and Normalization (VSN) in R[75]. This transformation coincides with the natural logarithm for the high intensities. Array data has been made available through the GEO data repository of the National Centre for Biotechnology Information under series GSE1574. Averaged (arithmetic mean) normalized intensities were calculated per gene per time point for *mdx *and control samples based upon 8 data points (2 biological replicates with 4 technical replicates each). This method is more efficient than ratio-based calculations for each hybridisation[76]. Genes were considered expressed when the average normalized intensity was higher than the background level. The background level was determined by calculating the averaged normalized intensity of 157 empty spots in all experiments plus 3 standard deviations.

Fold-changes in gene expression were calculated on a linear scale by subtraction of averaged normalized intensities of control samples from *mdx *samples at each time point, followed by returning *e *raised to the power of the difference. Maximum fold-changes per gene were determined according to the highest fold-change within the time course. Statistically significant differential gene expression per gene per time point was calculated between *mdx *and control samples by performing a two-tailed Student's *t*-test assuming equal distributions. Significance levels were set at 0.05 after applying a Bonferroni correction for multiple testing. Gene expression profiles were taken in consideration when at least one time point showed statistically significant differential gene expression between *mdx *and control samples.

Comparisons with other gene expression studies were facilitated by the program GeneHopper[77] that links annotations for different platforms.

### Clustering

Temporal differential gene expression profiles were scaled to time point t = 1 week. Selected profiles were grouped using k-means clustering into a predetermined number of clusters according to the correlation similarity measure (Spotfire DecisionSite 7.1.1, Functional Genomics package). Clustering was initiated using evenly spaced profiles as algorithm. This method generates profiles to be used as centroids that are evenly distributed between the minimum and maximum value for each variable in the selected profiles (from Spotfire DecisionSite User's Guide and Reference Manual).

### Comparison with data from Duchenne patients

In a previous study, gene expression levels in 2 pools of muscle RNA from 5 Duchenne patients (aged 5–6 years and aged 10–12 years, respectively) and 2 pools of muscle RNA from non-dystrophic controls (aged 5–12 years and aged 4–13 years, respectively), were evaluated on Affymetrix U95A and U95Av2 GeneChips^® ^[78]. We re-analysed the gene expression data to obtain expression levels for all genes, as well as the most recent annotation. To this end, publicly available cel-files  were loaded in Rosetta Resolver^® ^Gene Expression Analysis System v4.0 (Rosetta Biosoftware Inc., Seattle, WA). Data were processed and normalized with the Rosetta error model for Affymetrix U95A Genechips. Genes that showed differential expression (p < 0.001 and absolute fold-change >1.5) between the pools of dystrophic patients and non-dystrophic controls were exported and linked to mouse UniGene clusters with GeneHopper, based on HomoloGene annotation.

### Functional annotation

Gene Ontology annotation was developed by Compugen, using nomenclature obtained from the Gene Ontology consortium . Additional information was retrieved via OMIM and LocusLink .

### Quantitative RT-PCR

cDNA was synthesized by adding 40 ng of random hexamer primers to 1 μg of total RNA in a total volume of 11 μl. After denaturation for 10 min at 70°C and cooling for 10 min on ice, 4 μl of 5× first-strand buffer (MBI-Fermentas), 2 μl of 10 mM dNTPs and 2 μl (200 U/μl) RevertAid RNase H- (MBI-Fermentas) was added. The mixture was incubated for 10 min at room temperature 2 hours at 42°C. The cDNA synthesis was halted by heating at 70°>C for 10 min. Quantitative PCR assays, using 10 μl of 20× diluted cDNA, were run on a MyIQ real-time PCR detection system (BioRad), applying 36 cycles of 10 seconds denaturation (95°C), 20 seconds annealing (60°C), and 25 seconds extension (72°C). PCR mixtures contained 1× PCR Buffer (Roche), 3 mM MgCl_2_, 225 μM of each dNTP, 250 μg/ml BSA, 1× SYBR-Green (diluted from 10,000× stock, Molecular Probes), 10 nM fluorescein (BioRad), 2.5 U homemade Taq polymerase, 0.25 U AmpliTaq (Roche), and 10 pmol of forward and reverse primers (for sequences see Additional file 3). The PCR efficiencies, determined by analysis of a dilution series of a mixture of all cDNA samples over 5 orders of magnitude, ranged from 94.5% to 98% for the different primer pairs. Melting curves were analyzed to confirm single product formation. Gene expression levels were calculated using the gene expression macro provided by BioRad and normalized to glyceraldehyde-3-phosphate dehydrogenase (GAPDH, stable expression in all samples) expression levels.

## Abbreviations

DMD Duchenne muscular dystrophy

DGC Dystrophin-associated glycoprotein complex

NMJ Neuromuscular junction

VSN Variance stabilization and normalization

RT-PCR Reverse transcription followed by polymerase chain reaction

## Authors' contributions

RT performed the microarray hybridisations, analysed the microarrays and wrote the draft of the paper. ES helped to set up the microarray technique and assisted with the analysis. EdM was involved in the breeding of the mice, the isolation of the tissues, and the quantitative PCR experiments. GJvO edited the manuscript. JdD conceived the study. P'tH was involved in the microarray analysis and participated in the writing of the paper.

## Supplementary Material

Additional File 1 Averaged expression and significance levels for 1735 genes differentially expressed at at least one time point. Click here for file

Additional File 4 Costameric and DGC related gene expression. Click here for file

Additional File 5 Regeneration associated genes. Click here for file

Additional File 2 **Temporal loop design**. Hybridisations were done using a temporal loop design for the *mdx *and the control samples separately. The temporal loop design balances dyes and samples, and provides low variance between adjacent timepoints[73]. The order of age is maintained in the hybridisation scheme; each target is hybridised with the target of the following timepoint. Hybridisations are indicated by Hyb ID. Green and red boxes indicate labelling of target with Cy3 and Cy5 respectively. The number in the boxes indicates the age of the mouse. The boxes linked by an arrow are identical samples. Click here for file

Additional File 3 Primer sequences used for quantitative RT-PCR assays. Click here for file
