# Supplementary figures and images for: Muscle regeneration in dystrophin-deficient mdx mice studied by gene expression profiling
Source: BMC Genomics. 2005 Jul 13;6:98. doi: 10.1186/1471-2164-6-98 (PMC1190170; doi:10.1186/1471-2164-6-98)

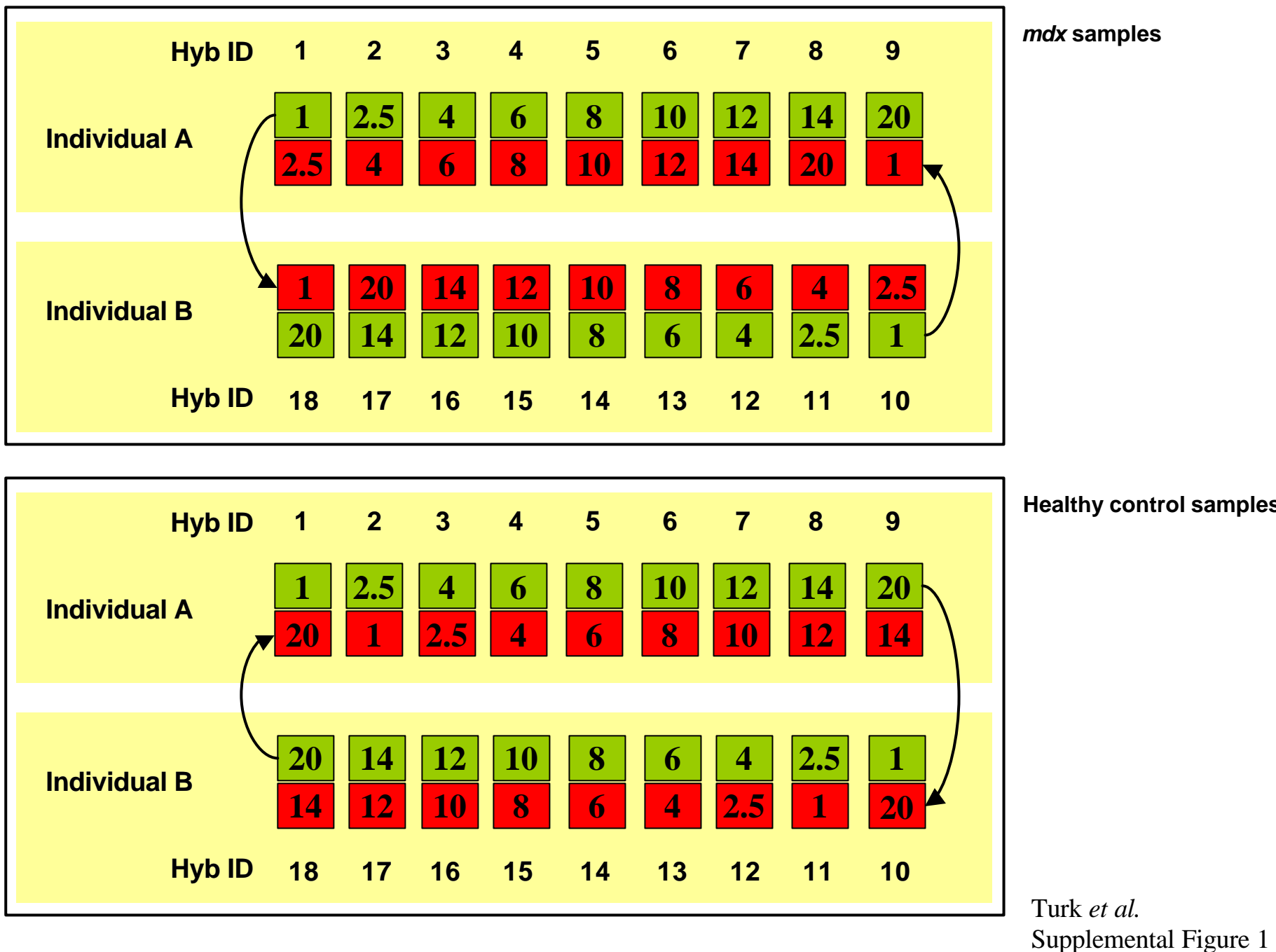

Supplement: Additional File 2 — Temporal loop design. Hybridisations were done using a temporal loop design for the mdx and the control samples separately. The temporal loop design balances dyes and samples, and provides low variance between adjacent timepoints[73]. The order of age is maintained in the hybridisation scheme; each target is hybridised with the target of the following timepoint. Hybridisations are indicated by Hyb ID. Green and red boxes indicate labelling of target with Cy3 and Cy5 respectively. The number in the boxes indicates the age of the mouse. The boxes linked by an arrow are identical samples. [file 1471-2164-6-98-S2.pdf]
